# How effector-specific is the effect of sequence learning by motor execution and motor imagery?

**DOI:** 10.1007/s00221-017-5096-z

**Published:** 2017-09-30

**Authors:** Jagna Sobierajewicz, Anna Przekoracka-Krawczyk, Wojciech Jaśkowski, Rob H. J. van der Lubbe

**Affiliations:** 1 Vision and Neuroscience Laboratory, NanoBioMedical Centre, Adam Mickiewicz University, Poznan, Poland; 2 Laboratory of Vision Science and Optometry, Faculty of Physics, Adam Mickiewicz University, Umultowska 85, 61-614 Poznan, Poland; 3 Institute of Computing Science, Poznan University of Technology, Poznan, Poland; 4 Cognitive Psychology and Ergonomics, University of Twente, Enschede, The Netherlands; 5 Department of Cognitive Psychology, University of Finance and Management, Warsaw, Poland

**Keywords:** Motor imagery, Motor execution, Learning, Execution mode, DSP task

## Abstract

The aim of the present study was twofold. First, we wanted to examine how effector specific the effect of sequence learning by motor execution is, and second, we wanted to compare this effect with learning by motor imagery. We employed a Go/NoGo discrete sequence production task in which in each trial a spatial sequence of five stimuli was presented. After a Go signal the corresponding spatial response sequence had to be executed, while after a NoGo signal, the response sequence had to be mentally imagined. For the training phase, participants were divided into two groups. In the index finger group, participants had to respond (physically or mentally) with the left or right index finger, while in the hand group they had to respond with four fingers of the left or right hand. In a final test phase both execution modes were compared and all trials had to be executed. Response times and the percentage of correct responses were determined to establish learning effects. Results showed that sequence learning effects as assessed in the test phase were independent of the effector used during the training phase. Results revealed the presence of aspecific learning effects in the case of learning a required motor task with an index finger, but sequence-specific learning effects, both due to motor execution and to motor imagery, were not effector specific.

**Electronic supplementary material:**

The online version of this article (doi:10.1007/s00221-017-5096-z) contains supplementary material, which is available to authorized users.

## Introduction

Fine motor skills involved in, for example, writing, drawing, or playing a musical instrument demand the production of relatively small movements, which are learned with a particular effector system (e.g., using the fingers, hands, feet, etc.) (Payne and Isaacs 1987; Keele et al. 1995). It has been argued that motor sequences can be acquired either in an effector-dependent manner (learning effects are limited to the trained effector system) or in an effector-independent manner (learning effects can be generalized to other effector systems) (Keele et al. 1995; Kovacs et al. 2009; Shea 2011). In the present study, we investigated how effector specific the effect of learning a sequential fine motor skill is. We examined sequence learning effects of motor execution and compared them with sequence learning effects of motor imagery.

Different views have been forwarded with respect to effector dependency of learned motor skills. For example, previous research showed that learning is effector dependent, which implies that training of one group of muscles does not generalize to another group of muscles (Bapi 2000; Verwey and Wright 2004; Osman 2005; Verwey and Clegg 2005). However, it has also been argued that motor skill learning is initially effector independent and may become effector dependent with extensive practice (Hikosaka et al. 1999). Results of Keele et al. (1995) indeed suggest that motor skill learning can be effector independent. Participants in a learning phase were asked to train a motor sequence with either three fingers (index-, middle-, and ring finger) or only with an index finger. In a transfer phase, participants were asked to switch; half of the participants continued to use the same effector system as in the learning phase, while the other half changed to the untrained motor system. Results revealed a comparable reduction in response time for all keys in both groups which supports the idea that motor skill learning is rather effector independent. Relevant to notice is that effects concerned all keys of the motor sequence. The first key of a motor sequence is generally thought to reflect the time to initiate the sequence, while the subsequent keys are more specific to execution (Abrahamse 2013). Thus, learning effects transferred both with regard to initiation and execution of the relevant sequence.

Verwey and Wright (2004) examined sequence learning with one group of participants that trained with three fingers of one hand, and another group that trained with three fingers of two hands. In the subsequent test phase, participants of both groups executed familiar and unfamiliar sequences in the same manner as during practice and also with the hand configuration of the other group. Results for familiar sequences revealed slower execution for the unpracticed as compared with the practiced hand configuration, suggesting effector-specific learning. However, execution of familiar sequences with the unpracticed hand was still faster than execution of new (unfamiliar) sequences. These findings suggest that the learned sequence representation consists of both effector-independent and effector-dependent components.

The notion that motor skill learning transfers from an effector-independent stage to an effector-dependent stage may be due to the involvement of representations at two different levels. Initially, representations may develop at a cognitive level that, for example, contain spatio-temporal characteristics of the movement, while later representations develop at a motor level that are muscle specific and concern more detailed characteristics of the movement (e.g., see Verwey 2016). This cognitive level seems related to the idea of a motor program (i.e., an abstract representation of a movement that organizes the performance of motor actions including their spatio-temporal aspects) (Schmidt 1975). It has been proposed that the two types of representations (i.e., cognitive and motor) develop separately (Shea 2011). Furthermore, these two representational levels have been related with different neural mechanisms (see Hikosaka et al. 1999; Hikosaka et al. 2002), which will be further detailed in the discussion. The results obtained by Keele et al. (1995) may thus be understood as due to the development of representations at a cognitive level that can be easily transferred to an unpracticed effector system. On the other hand, the results from the study of Verwey and Wright (2004) support also the development of representations at a motor level but this requires more extensive practice.

In our earlier research, we were especially interested in the learning of a fine sequential motor skill by motor imagery (Sobierajewicz et al. 2016, 2017), which can be defined as the mental simulation of a movement without producing an overt action (Jeannerod 2001). A question that emerged from our studies was whether learning by motor imagery involves a cognitive and/or a motor level. It is well known that motor skills can be acquired not only by repeating (i.e., physical practice) a particular movement, but also by motor imagery (Jeannerod 2001; Allami et al. 2008; Doussoulin and Rehbein 2011). It has additionally been argued that motor imagery relies on similar processes and obeys the same rules as motor execution (e.g., with regard to the timing and brain mechanisms underlying motor imagery and motor execution (Decety et al. 1989; Xu et al. 2014; Sobierajewicz et al. 2016, 2017). However, the effect of motor imagery on sequence learning is not as strong as the effect of physical practice (Feltz and Landers 1983; Hird et al. 1991; Gentili 2006; Gentili et al. 2010; Schuster et al. 2011; Debarnot et al. 2015; Gentili and Papaxanthis 2015; Sobierajewicz et al. 2016), indicating that motor execution is more effective in acquisition of a motor skill. Although previous findings indicate that motor imagery improves the accuracy and may enhance the speed of a movement (Sobierajewicz et al. 2016), the relevance of the execution mode while learning a fine motor skill (i.e., small finger or hand movements such as writing, tapping or drawing) with motor imagery was not yet examined.

A first issue to be addressed in the present study is whether the observations made by Keele et al. (1995), which pointed to the involvement of representations at a cognitive level, can be replicated in a slightly different paradigm that we used to examine the learning of a sequential motor skill by motor imagery. We employed a Go/NoGo DSP task wherein in each trial a spatial sequence of five stimuli was presented, which had to be next either executed or mentally simulated. A second issue to be addressed is whether the evidence for the involvement of representations at a cognitive level also applies to the learning of a motor skill with motor imagery. Two execution (and motor imagery) modes were employed. One group had to practice movement sequences physically (or mentally) using the index fingers, while a second group had to practice using four fingers of the left or right hand. Based on the ideas presented in the introduction, the following predictions can be made. If representations develop at a cognitive level and then transfer from the practiced effector system to the unpracticed effector system is possible, in that case, sequence learning, either by execution or by motor imagery, is effector independent. If representations develop at a motor level, then no transfer is possible. The possible transfer can be examined in a final test phase in which familiar executed, familiar imagined, and unfamiliar movement sequences have to be carried out, either in the same mode as during practice, or in a different mode.

## Methods

### Participants

Twenty-four participants (eight males, sixteen females) recruited from the Adam Mickiewicz University took part in our experiment. All participants were aged between 20 and 28 years (*M*_age_ = 23.6, SD 2.34). The inclusion criteria for participation were absence of any mental or neurological disorder. Informed consent was obtained from each participant prior to the start of the experiment. Participants were requested to complete Annett's Handedness Inventory (Annett 1970). Twenty-free of them were right-handed, and one of them was left-handed. All participants were naïve as to the purpose of the experiment. The study was approved by the local Ethics Committee of the Adam Mickiewicz University and was performed in accordance with the Declaration of Helsinki.

### Stimuli and task

**Fig. 1:**
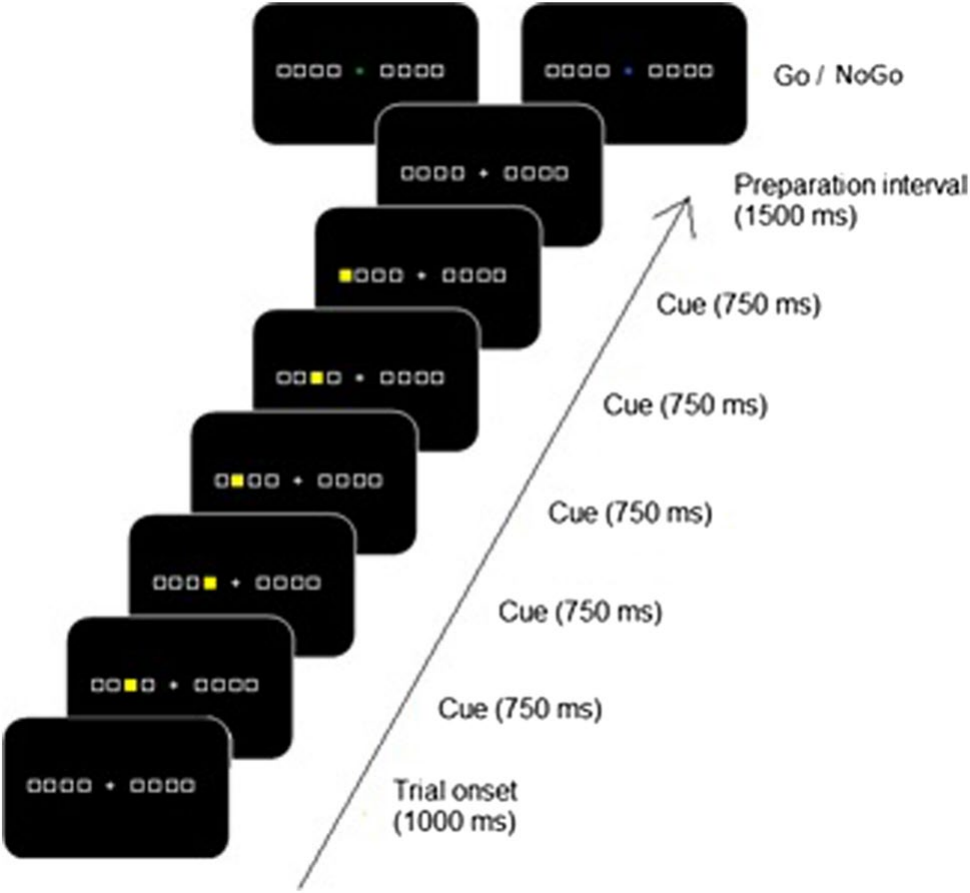
An overview of stimulus presentation in the Go/NoGo discrete sequence production (DSP) task. Two possible informative cues were presented: a green cross implied that the sequence had to be executed (Go signal) while a blue cross indicated that execution of the sequence had to be mentally imagined (NoGo signal)

A trial consisted of the presentation of a sequence of five visual stimuli. An example of a stimulus sequence is presented in Fig. 1. Stimuli were displayed on a CRT monitor with a refresh rate of 60 Hz. Each trial started with a beep of 300 Hz for 300 ms. A fixation cross (1.3°) was presented at the center of the screen with eight horizontally aligned squares (2.5°)—four on the left and four on the right side of the fixation cross. Each square was assigned to a button on the keyboard (*a*, *s*, *d*, *f* keys and the ;, *l*, *k*, *j* keys). The eight stimulus squares had a total visual angle of 26.5°. The fixation cross and the eight squares were drawn with a gray color line on a black background. After a time interval of 1000 ms, one of the squares was filled yellow for 750 ms, a second square was filled, etc., until a fifth square was filled. The stimulus sequence always appeared on either the right or the left side of fixation. An informative cue (a cross) appeared after a preparation interval of 1500 ms relative to the offset of the last square. The cue was presented in one out of two possible colors. A green cross indicated that the cued response sequence had to be executed (a Go signal). A blue cross implied that the response sequence had to be mentally imagined (a NoGo signal). Participants should imagine executing the five spatially corresponding key presses in the same order as the stimulus sequence. They were also asked to execute or imagine executing the required sequence as fast and accurately as possible. All participants were requested to concentrate on the fixation cross during the presentation of the sequence and while carrying out the task.

### Procedure

At the start of the experiment each participant received verbal instructions. Participants sat in a dimly lit room at a viewing distance of 70 cm from a screen. The experiment was conducted on two consecutive days (2 h per day) to increase the number of trials. The measurement only on day 1 would last too long for participants and the effect could be distracted due to tiredness. On the first day, participants performed five training blocks. On the second day, two additional training blocks had to be carried out (40 sequences had to be executed and 40 sequences had to be imagined in each block, with the same number of repetitions for the right and for the left hand), and after a break two final test blocks had to be executed (40 sequences executed before; 40 sequences imagined before; 40 totally new sequences). To eliminate finger-specific effects for the hand condition and to ensure that all sequences had the same level of complexity, we employed different sequences, which are shown in the Appendix. We used six different structures (12432, 13423, 14213, 13241, 14312, and 21431) with four different versions of sequences per structure. We counterbalanced the sequences across participants and across fingers.

Participants were assigned to an index finger group (*n* = 12) or to a hand group (*n* = 12). In the index finger group, sequences had to be carried out in the training phase either physically or mentally using the index finger of their left and right hand. In the hand group, participants had to execute or imagine sequences using four fingers (little finger, ring finger, middle finger and index finger) of their left and right hand. In the case of mental execution, all participants were instructed to use a first-person perspective (i.e., to imagine the sensation of executing a sequence), i.e., they had to use kinesthetic motor imagery. To ensure that all participants understood the required task and the difference between visual vs. motor imagery, participants were given two examples (“imagine yourself walking on the street—you can see yourself walking”/“imagine as if you are walking—you imagine your movements during walking”, respectively). In the test phase, six participants from the index finger group executed sequences in the first block using four fingers of the left or right hand, and in the second block they only used the index fingers; the other six participants received these blocks in a reversed order (see Table 1). Similarly, in the hand group, six participants executed the sequences in the first test block using four fingers of the left or right hand, and in the second block they only used the index fingers; the other six participants had these blocks in a reversed order. In the case of executing sequences with four fingers, all participants placed their little finger, ring finger, middle finger and index finger of their left and right hand, respectively, on the *a*, *s*, *d*, *f* keys and the ;, *l*, *k*, *j* keys.

**Table 1:** An overview of the execution mode for the index finger group and for the hand group in the training phase and the test phase

Group	The training phase	The test phase	
	Block 1–7	Block 1	Block 2
Index finger group (*n* = 12)	Index finger (*n* = 12)	Index finger (*n* = 6) Four fingers (*n* = 6)	Four fingers (*n* = 6) Index finger (*n* = 6)
Hand group (*n* = 12)	Four fingers (*n* = 12)	Index finger (*n* = 6) Four fingers (*n* = 6)	Four fingers (*n* = 6) Index finger (*n* = 6)

Four fingers implies executing the sequence with the little finger, ring finger, middle finger and index finger

Halfway each block and after each block a pause was provided during which the participant could relax. After completion of each block, participants were shown their mean reaction times and error percentages. Moreover, feedback about incorrect responses was given after the end of response only when a participant pressed the button before a Go/NoGo signal or in the case of a false response sequence.

Electroencephalographic (EEG) activity was also measured in this study, but we decided not to focus on these results (i.e., comparing EEG activity between groups during motor preparation in the training phase) as they seem beyond the scope of the current manuscript.

### Behavioral parameters

Response time (RT) was defined as the time between onset of the Go signal and depression of the first key, and as the time between two consecutive key presses within a sequence (De Kleine and Van der Lubbe 2011; Ruitenberg et al. 2011). To reduce the number of levels of the Key variable, we averaged RTs of keys 2–5, which results in two levels, one including the first key press, and the second including the averages of keys 2–5 (Sobierajewicz et al. 2016). In the training phase, mean RTs were statistically evaluated by a repeated measures analysis of variance (ANOVA) with Block (7), and Key (2) as within-subject factors, and Group (2) as between-subject factor. ANOVAs in the test phase were performed with Type of Sequence (3: familiar executed, familiar imagined, unfamiliar), and Key (2) as within-subject factor, and Group (2) and Execution Mode (2) as between-subject factors. This was done separately for the first and the second block as sequence-specific learning effects might no longer be detectable in the second test block due to learning the new sequences.

Analyses on PCs were carried out after performing an arcsine transformation to stabilize variances (Abrahamse and Verwey 2008). A repeated measures ANOVA was run for the training phase with Group (2) as between-subject factor, and Block (7) as within-subject factor; and in the test phase for each block separately with Group (2), Execution Mode (2) as between-subject factors, and Type of Sequence (3) as within-subject factor.

### EMG

Electromyographic activity (EMG) was measured to establish whether participants correctly executed the required task, i.e., in the case of the motor execution they should flex their muscles, while in the case of motor imagery they should not. EMG was measured bipolarly by attaching EMG electrodes on the musculus flexor digitorum superficialis and on the processus styloideus ulnae of the right and left hand. EMG from both hands was recorded with Vision Recorder (Brain Products—version 2.0.3) with a sampling rate of 1000 Hz. Offline, analyses were performed with Brain Vision Analyzer (version 2.0.4) software. The signal was low-pass filtered at 50 Hz (24 dB/oct) and high-pass filtered at 20 Hz (24 dB/oct). The threshold for a movement was set at 60–160 µV depending on the resting level of the individual participant. Wavelet analyses were performed to determine the extent of motor activation of a required motor task. A complex Morlet wavelet was chosen (*c* = 5) with the lower and upper boundaries for the extracted layer set at 20 and 50 Hz.

After logarithmic transformation, the results of the wavelet analysis were analyzed with the following factors: Group (2), Block (7), EMG channel (right relevant hand, left relevant hand (2), and Type of Sequence (2—motor imagery/motor execution). We choose a time window from a Go/NoGo signal until 6000 ms, as this time window seems sufficient to execute or imagine the required type of sequence.

All statistical analyses were performed with SPSS (IBM Statistics SPSS 24). The level of significance was set at *p* < 0.05. Greenhouse–Geisser *ɛ* correction was applied whenever appropriate. We examined linear, quadratic and cubic contrasts to increase sensitivity for detecting gradual differences as a function of Block.

## Results

### Training phase

**Fig. 2:**
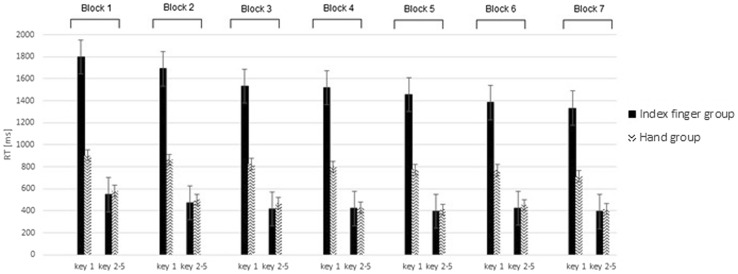
Mean response times (RTs) in milliseconds (ms) in the training phase as a function of Key. Error bars represent standard errors

RT results for the training phase are presented in Fig. 2. Results revealed faster RTs for the hand group than for the index finger group, *F*(1,22) = 16.16, *p* = 0.001, *η*_*p*_^2^ = 0.42. RTs changed as a function of Block, *F*(6,132) = 30.98, *ϵ* = 0.38, *p* < 0.001, *η*_*p*_^2^ = 0.59. Contrast analyses revealed a linear trend, *F*(1,22) = 75.28, *p* < 0.001, and a quadratic trend, *F*(1,22) = 8.12, *p* < 0.001, suggesting that these effects reflect a general decrease in RT during practice, while this decrease seems to be stronger in the earliest blocks. A nearly significant interaction between Block and Group was observed, *p* = 0.06, indicating that RTs for the index finger group tended to decrease more across blocks than RTs for the hand group.

A main effect of Key was observed, *F*(1,22) = 112.1, *p* < 0.001, *η*_*p*_^2^ = 0.84 (mean RT for key 1 and the average of keys 2–5 in the index finger group, respectively, 1531, 439 ms; in the hand group: 807, 466 ms, respectively). An interaction between Key and Group was observed, *F*(1,22) = 30.78, *p* < 0.001, *η*_*p*_^2^ = 0.58. Inspection of Fig. 2 clearly shows that the first key press in the index group was executed slower than the first key press in the hand group. Separate *t* tests confirmed this effect, *t*(11) = 4.19, *p* = 0.002. This observation may indicate that the time needed to initiate a sequence was longer for the index finger group than for the hand group. The average RTs of keys 2–5 was similar in both groups, *t*(11) = 0.58, *p* = 0.57. An interaction between Block and Key was observed, *F*(6,132) = 4.79, *ϵ* = 0.39, *p* = 0.009, *η*_*p*_^2^ = 0.18. Separate tests revealed that RTs for the first key press changed as a function of Block, *F*(6,132) = 15.46, *ϵ* = 0.37, *p* < 0.001, *η*_*p*_^2^ = 0.41. A significant interaction between Block and Group was observed, *p* < 0.05, indicating that RTs for the first key press for the index finger group decreased more across blocks than RTs for the hand group. RTs for the average of keys 2–5 also changed as a function of Block, *F*(6,132) = 39.87, *ϵ* = 0.5, *p* < 0.001, *η*_*p*_^2^ = 0.64. No significant interaction between Block and Group was observed, *p* = 0.51, showing similar decrease of RTs in the case of both groups. We also observed an interaction between Block, Key and Group, *F*(6,132) = 3.04, *p* = 0.05, *η*_*p*_^2^ = 0.12, showing a stronger decrease of mean RT for the first key press for the index finger group as compared with the hand group with practice.

**Fig. 3:**
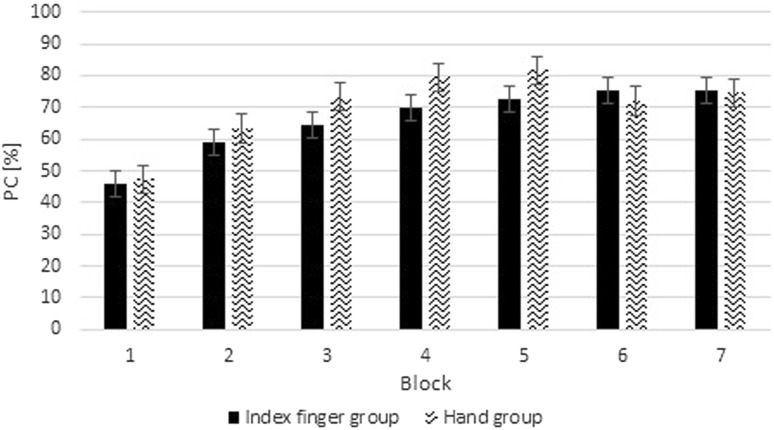
Percentage of correct response (PC) of the averages of to-be-executed sequences in the training phase for the index finger group (IG) and the hand group (HG). Error bars represent standard errors

A repeated-measure ANOVA was also performed on arcsin transformed error percentages as a function of Group (2) and Block (7). Results revealed no significant differences in accuracy between groups, *F*(1,22) = 0.39, *p* = 0.54, *η*_*p*_^2^ = 0.02. A main effect of Block was observed, *F*(6,132) = 26.9, *ϵ* = 0.57, *p* < 0.001, *η*_*p*_^2^ = 0.55 (linear trend: *F*(1,22) = 56.49, *p* < 0.001; quadratic trend: *F*(1,22) = 27.91, *p* < 0.001) (Fig. 3). These results indicate that the number of correct responses increased with practice and this effect was most prominent in the earlier blocks. No significant interaction between Block and Group was observed, *p* = 0.19. Thus, these results showed that the number of correct responses increased with practice in both groups.

### Test phase

For the test phase, analyses were performed for RTs for each block separately with the factors Group (2), Execution Mode (2, four fingers/index fingers), Type of Sequence (3), and Key (2). A repeated-measure ANOVA was also performed on arcsin transformed error percentages as a function of Group (2), Execution Mode (2), and Type of Sequence (3) for each block of the test phase.

### RT

**Fig. 4:**
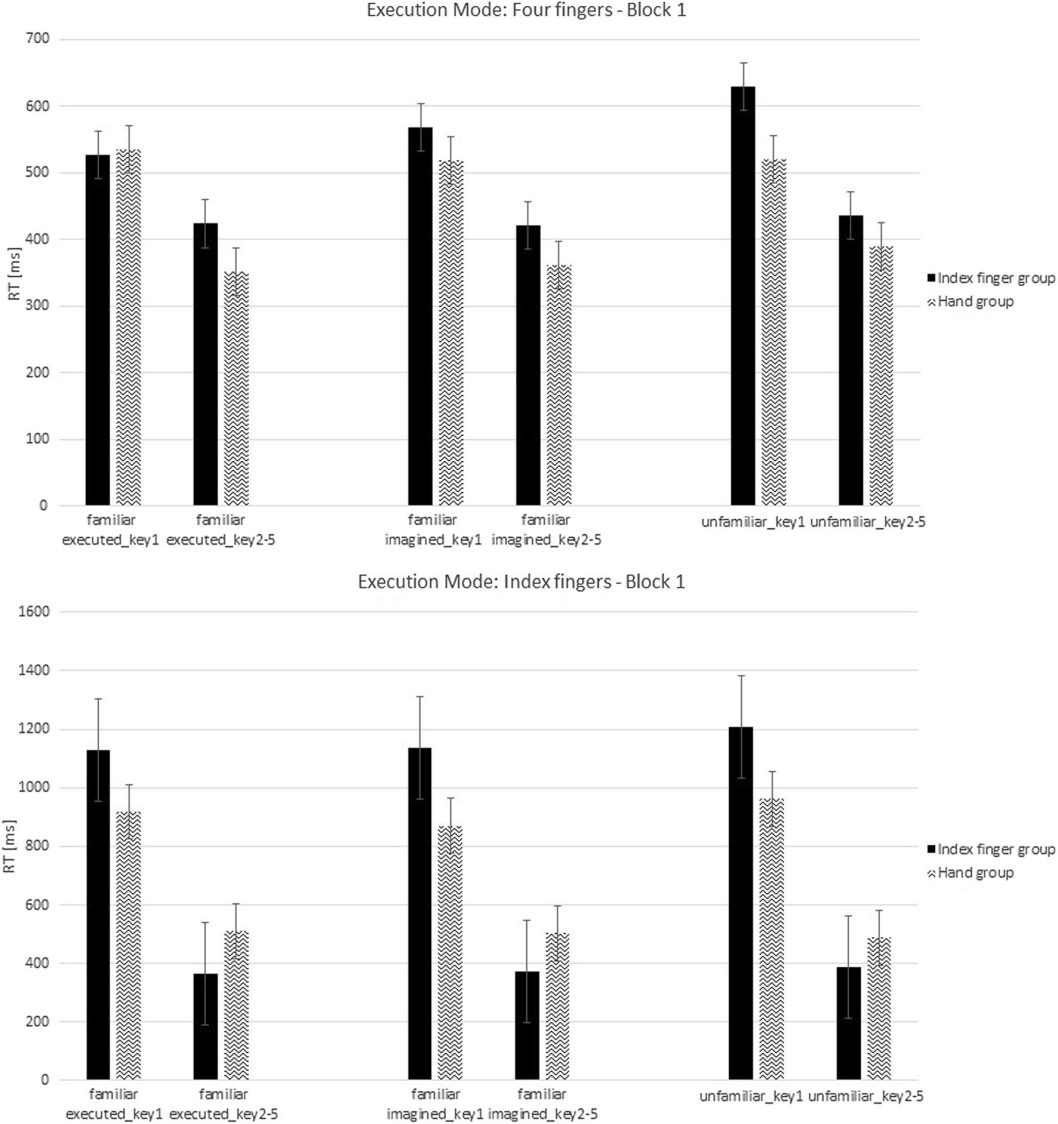
Response times (RTs) in milliseconds from the first block in the test phase for sequences, which were executed with four fingers and only with an index finger presented for each type of sequence. Different scales were used to emphasize the differences between groups. Error bars represent standard errors

The analysis for the first block revealed no significant difference in mean RT between groups, *F*(1,20) = 0.91, *p* = 0.32, *η*_*p*_^2^ = 0.05 (Fig. 4). A significant difference was observed as a function of Execution Mode, *F*(1,20) = 23.36, *p* < 0.001, *η*_*p*_^2^ = 0.54, indicating that sequences were executed faster with four fingers than with the index finger (Fig. 4). These results demonstrate that the use of different execution modes affect mean RTs, which we also observed in the training phase. No significant interaction between Group and Execution Mode was observed, *F*(1,20) = 0.001, *p* = 0.98, *η*_*p*_^2^ < 0.001. These results might indicate that motor execution in the test phase did not depend on the execution mode in the training phase (but see below). A main effect of Type of Sequence was observed, *F*(2,40) = 4.2, *ϵ* = 0.99, *p* = 0.02, *η*_*p*_^2^ = 0.17. Separate *t* tests revealed that unfamiliar sequences were executed slower than familiar executed sequences, *t*(23) = 2.4, *p* < 0.03, and also slower than familiar imagined sequences, *t*(23) = 2.81, *p* = 0.01. No difference in RT was observed between familiar executed and familiar imagined sequences, *p* = 0.91. These findings indicate that both motor execution and motor imagery in the training phase induced sequence-specific learning effects. No significant interaction between Type of Sequence and Group was observed, *F*(2,40) = 1.36, *p* = 0.27, *η*_*p*_^2^ = 0.06, and no significant interaction between Type of Sequence and Execution Mode was observed, *F*(2,40) = 0.28, *p* = 0.76, *η*_*p*_^2^ = 0.01. No significant interaction between Type of Sequence × Group × Execution Mode was observed, *F*(2,40) = 0.07, *p* = 0.93, *η*_*p*_^2^ = 0.003. The absence of interactions between Type of Sequence and the factor Group suggests that the observed sequence learning effects in the Test phase do not depend on the execution mode in the Training phase.

A main effect of Key was observed, *F*(1,20) = 69.44, *p* < 0.001, *η*_*p*_^2^ = 0.78, indicating that first key presses were slower than the average of key presses 2–5. No significant interaction between Key and Group was observed, *F*(1,20) = 3.91, *p* = 0.06, *η*_*p*_^2^ = 0.16, but a significant interaction was observed between Key and Execution Mode, *F*(1,20) = 24.53, *p* < 0.001, *η*_*p*_^2^ = 0.55. A significant interaction between Key × Group × Execution Mode was observed, *F*(1,20) = 4.3, *p* = 0.05, *η*_*p*_^2^ = 0.18. This observation suggests that motor execution in the test phase may depend on the execution mode in the training phase, either for the first key press, or for the average of key 2–5 (see below). Furthermore, a significant interaction between Type of Sequence and Key was observed, *F*(2,40) = 69.44, *p* < 0.001, *η*_*p*_^2^ = 0.78. No significant interaction between Type of Sequence × Key × Group was observed, *F*(2,40) = 1.13, *p* = 0.33, *η*_*p*_^2^ = 0.05, and no significant interaction between Type of Sequence × Key × Execution Mode was observed, *F*(2,40) = 1.19, *p* = 0.31, *η*_*p*_^2^ = 0.05. To clarify the aforementioned interactions, whether they concerned the first key or the average of keys 2–5, we performed separate analyses for each level of the factor Key with Type of Sequence (3), Group (2), and Execution Mode (2) as factors.

For the first key, a main effect of Type of Sequence was observed, *F*(2,40) = 3.54, *ϵ* = 0.93, *p* = 0.04, *η*_*p*_^2^ = 0.15. Separate *t* tests revealed that the first key press for familiar executed sequences was executed faster than the first key press for unfamiliar sequences, *t*(11) = 2.04, *p* = 0.05; the first key press for familiar imagined sequences was also executed faster than the first key press for unfamiliar sequences, *t*(11) = 2.83, *p* = 0.009. No group differences were observed for the first key, *F*(1,20) = 2.32, *p* = 0.14. A significant difference was observed as a function of Execution Mode, *F*(1,20) = 26.14, *p* < 0.001, indicating that the first key press was executed faster with four fingers than with the index finger (see above).

For the average of keys from 2 to 5, we observed a significant interaction between Group and Execution Mode, *F*(1,20) = 8.99, *p* = 0.007. Separate *t* tests revealed no significant difference in pressing the keys with four fingers between the index finger group and the hand group, *t*(10) = 1.31, *p* = 0.22; in the case of executing a motor sequence only with the index fingers, participants in the index finger group pressed the keys from 2 to 5 faster relative to the hand group, *t*(10) = 3.0, *p* = 0.01. These results show aspecific learning effects of execution mode used in the training phase by participants from the index finger group.

**Fig. 5:**
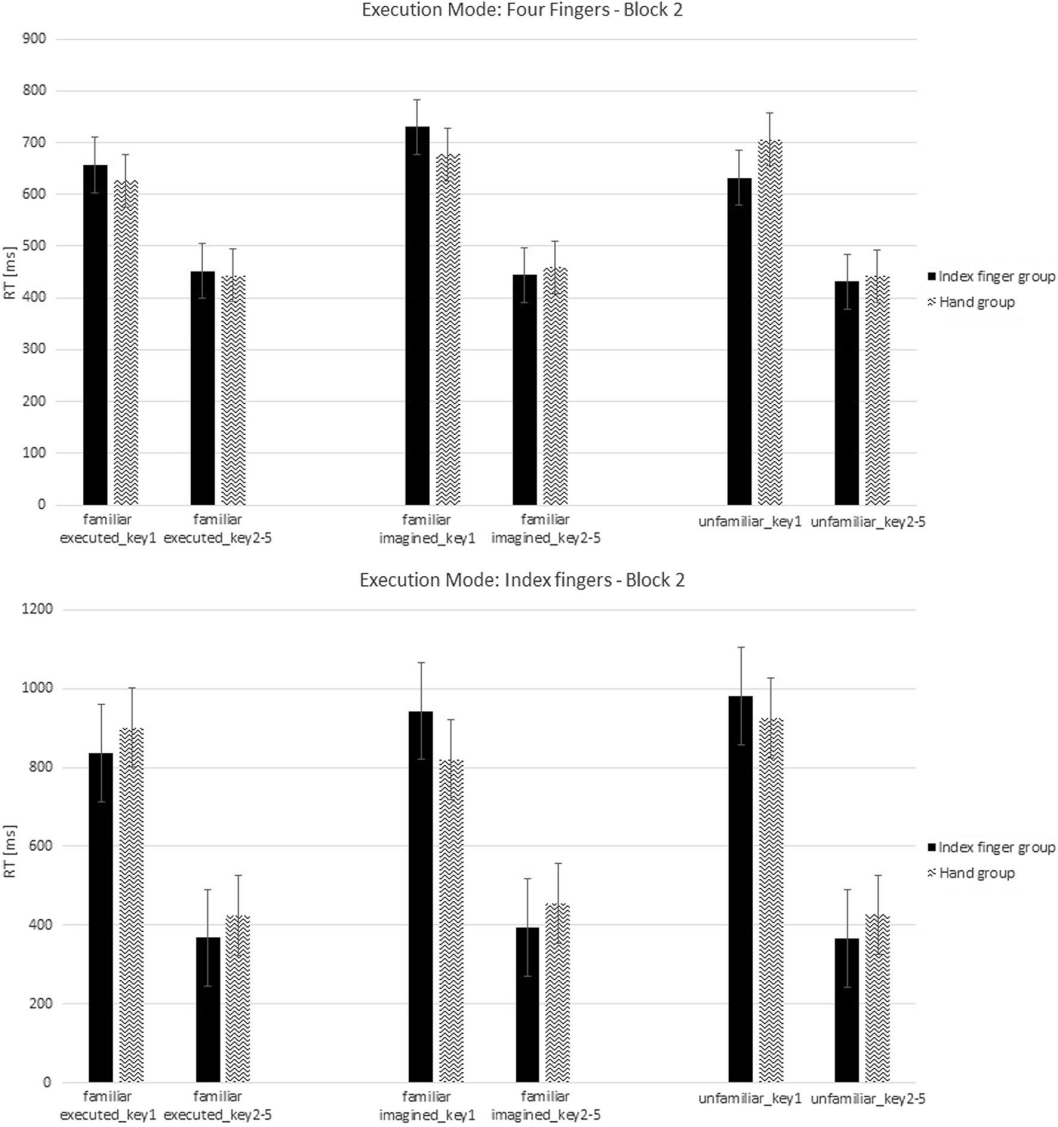
Response times (RTs) in milliseconds from the second block in the test phase for sequences, which were executed with four fingers and only with an index finger presented for each type of sequence. Different scales were used to emphasize the differences between groups. Error bars represent standard errors

The RT analysis for the second block of the test phase revealed no effect of Group, *F*(1,20) = 0.01, *p* = 0.92, *η*_*p*_^2^ = 0.001 (Fig. 5). Furthermore, no significant interactions were observed between Group and Execution Mode, *F*(1,20) = 0.006, *p* = 0.94, *η*_*p*_^2^ < 0.001; between Type of Sequence and Group, *F*(2,40) = 0.81, *p* = 0.43, *η*_*p*_^2^ = 0.04; between Type of Sequence and Execution Mode, *F*(2,40) = 0.64, *p* = 0.5, *η*_*p*_^2^ = 0.03. No significant interaction between Type of Sequence × Group × Execution Mode was observed, *F*(2,40) = 1.08, *p* = 0.34, *η*_*p*_^2^ = 0.05. A main effect of Key was observed, *F*(1,20) = 37.9, *p* < 0.001, *η*_*p*_^2^ = 0.66, indicating that the first key press was slower than the average of key presses from 2 to 5. No significant interaction between Key and Group was observed, *F*(1,20) = 0.2, *p* = 0.66, *η*_*p*_^2^ = 0.01, but a significant interaction was observed between Key and Execution Mode, *F*(1,20) = 5.31, *p* = 0.03, *η*_*p*_^2^ = 0.21, which is detailed below. The interaction between Type of Sequence and Key was not significant, *F*(2,40) = 1.29, *p* = 0.28, *η*_*p*_^2^ = 0.06. No significant interactions were observed between Type of Sequence × Key × Group, *F*(2,40) = 1.37, *p* = 0.26, *η*_*p*_^2^ = 0.06, and between Type of Sequence × Key × Execution Mode, *F*(2,40) = 1.25, *p* = 0.29, *η*_*p*_^2^ = 0.06.

Separate analyses for each level of the factor Key revealed a significant difference of Execution Mode, *F*(1,20) = 5.1, *p* < 0.04, for the first key press, which showed again that the first key press was faster with four fingers than with the index finger. For the average of keys from 2 to 5, no significant differences were observed as a function of Group and Execution Mode, *p* < 0.05.

### PC

**Fig. 6:**
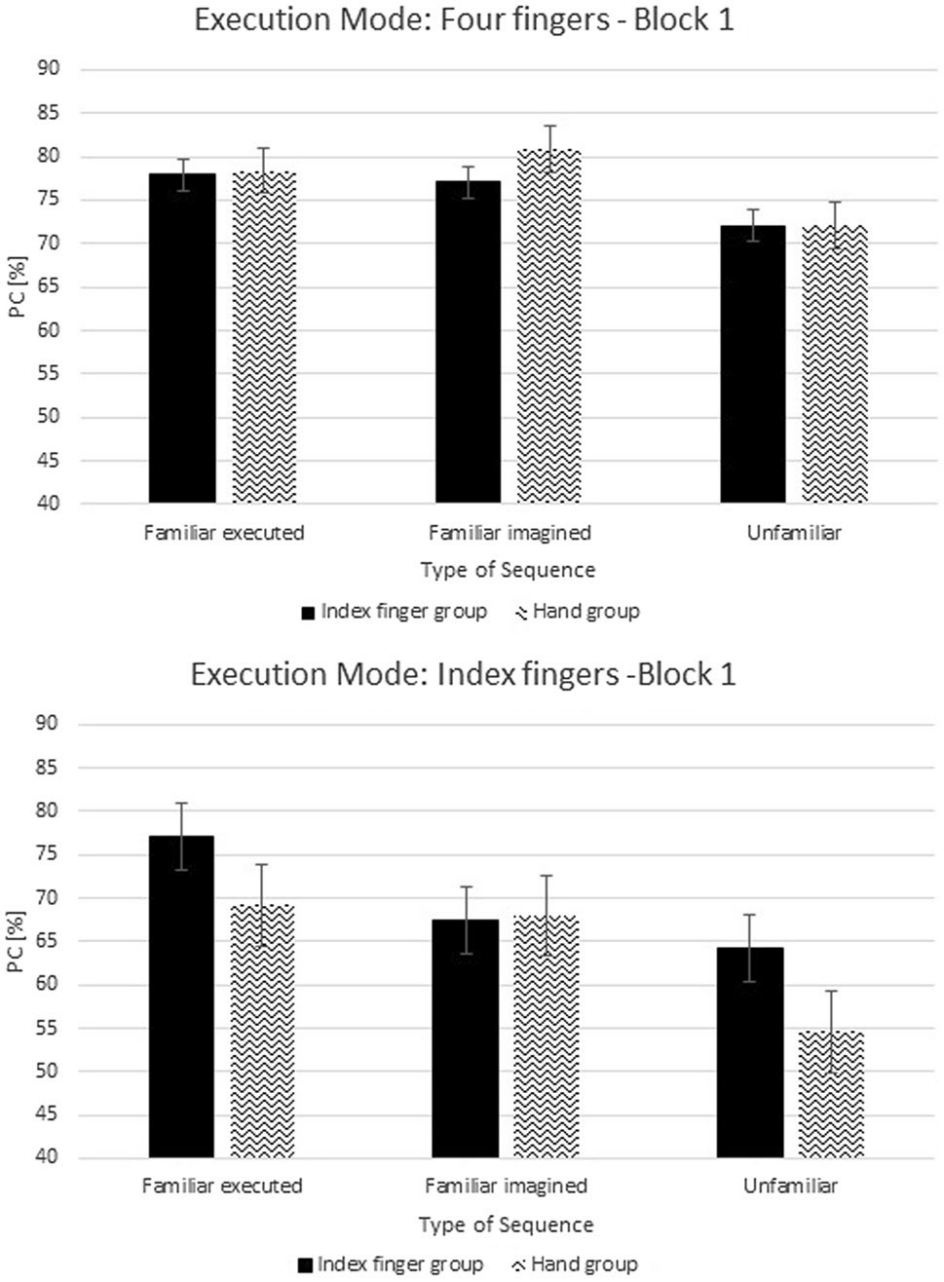
Percentage of correct responses (PC) from the first block in the test phase for sequences, which were executed with four fingers and with an index finger. Error bars represent standard errors

A repeated-measure ANOVA was performed on PC with the factors Group (2), Execution Mode (2), and Type of Sequence (3) for each block of the test phase. Results for the first block of the test phase revealed no significant difference in PC between groups, *F*(1,20) = 0.09, *p* = 0.76, *η*_*p*_^2^ = 0.005, and no significant difference as a function of Execution Mode, *F*(1,20) = 2.6, *p* = 0.12, *η*_*p*_^2^ = 0.12 (Fig. 6). Furthermore, no significant interaction between Group and Execution Mode was observed, *F*(1,20) = 0.32, *p* = 0.58, *η*_*p*_^2^ = 0.02. These results indicate that accuracy of performance was not dependent on the execution mode in the training phase, which differed between the groups, not dependent on the execution mode in the test phase, and not dependent on whether the execution mode changed or initially stayed the same in test phase. A main effect of Type of Sequence was observed, *F*(2,40) = 7.4, *ϵ* = 0.97, *p* = 0.002, *η*_*p*_^2^ = 0.27. Separate *t* tests revealed that unfamiliar sequences were executed less accurately than familiar executed sequences, *t*(23) = 3.69, *p* = 0.001, and unfamiliar sequences were executed less accurately than familiar imagined sequences, *t*(23) = 2.67, *p* = 0.01; no difference between familiar executed and familiar imagined sequences was observed, *p* = 0.34. No significant interaction between Type of Sequence and Group was observed, *F*(2,40) = 1.01, *p* = 0.37, *η*_*p*_^2^ = 0.05; and no significant interaction between Type of Sequence and Execution Mode was observed, *F*(2,40) = 1.11, *p* = 0.34, *η*_*p*_^2^ = 0.05. The interaction between Type of Sequence × Group × Execution Mode was also not significant, *F*(2,40) = 0.22, *p* = 0.8, *η*_*p*_^2^ = 0.01.

**Fig. 7:**
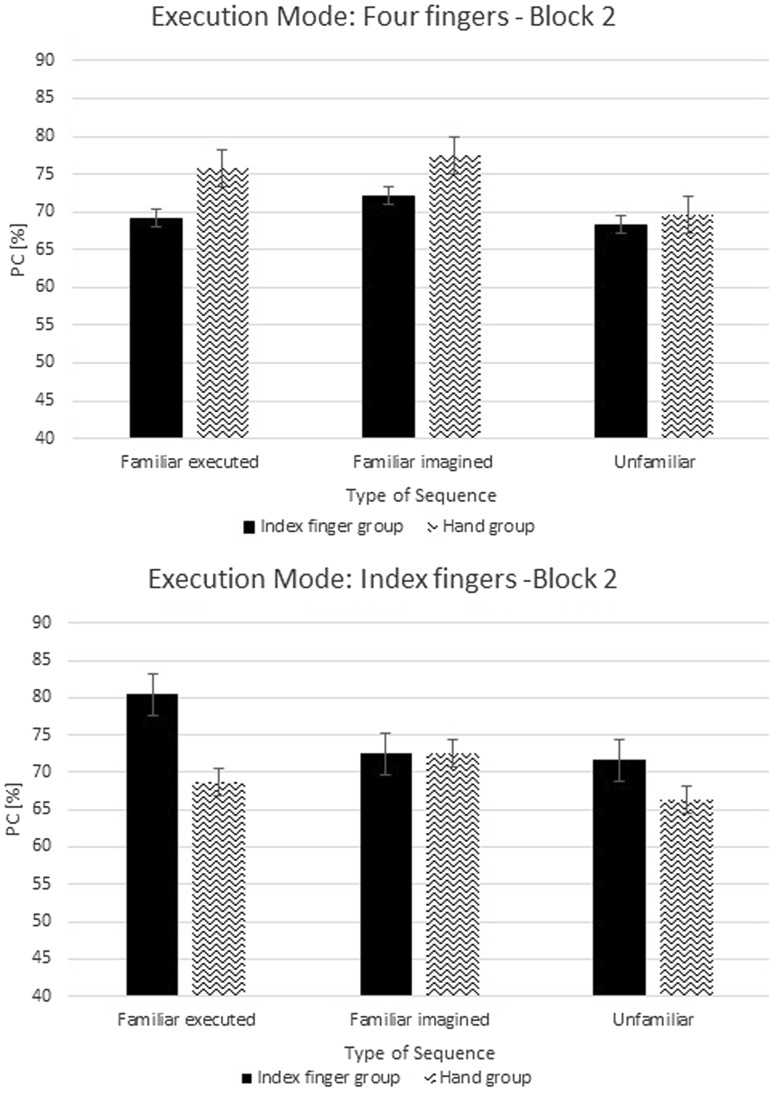
Percentage of correct responses (PC) from the second block in the test phase for sequences, which were executed with four fingers and only with an index finger. Error bars represent standard errors

Results for the second block of the test phase revealed no significant difference in PC between groups, *F*(1,20) < 0.001, *p* = 0.99, *η*_*p*_^2^ < 0.001, and no significant difference as a function of Execution Mode was observed, *F*(1,20) < 0.001, *p* = 0.99, *η*_*p*_^2^ < 0.001 (Fig. 7). No significant interaction between Group and Execution Mode was observed, *F*(1,20) = 0.39, *p* = 0.54, *η*_*p*_^2^ = 0.02. These findings again indicate that accuracy of performance was not dependent on the execution mode in the training phase, and also not dependent on the execution mode in the test phase, and not dependent on the change of execution mode in this second block in the test phase. No main effect of Type of Sequence was observed, *F*(2,40) = 0.8, *ϵ* = 1.0, *p* = 0.46, *η*_*p*_^2^ = 0.04. No significant interactions were observed between Type of Sequence and Group, *F*(2,40) = 0.3, *p* = 0.74, *η*_*p*_^2^ = 0.02; between Type of Sequence and Execution Mode, *F*(2,40) = 0.32, *p* = 0.73, *η*_*p*_^2^ = 0.02; and between Type of Sequence × Group × Execution Mode, *F*(2,40) = 0.63, *p* = 0.54, *η*_*p*_^2^ = 0.03.

### EMG

**Fig. 8:**
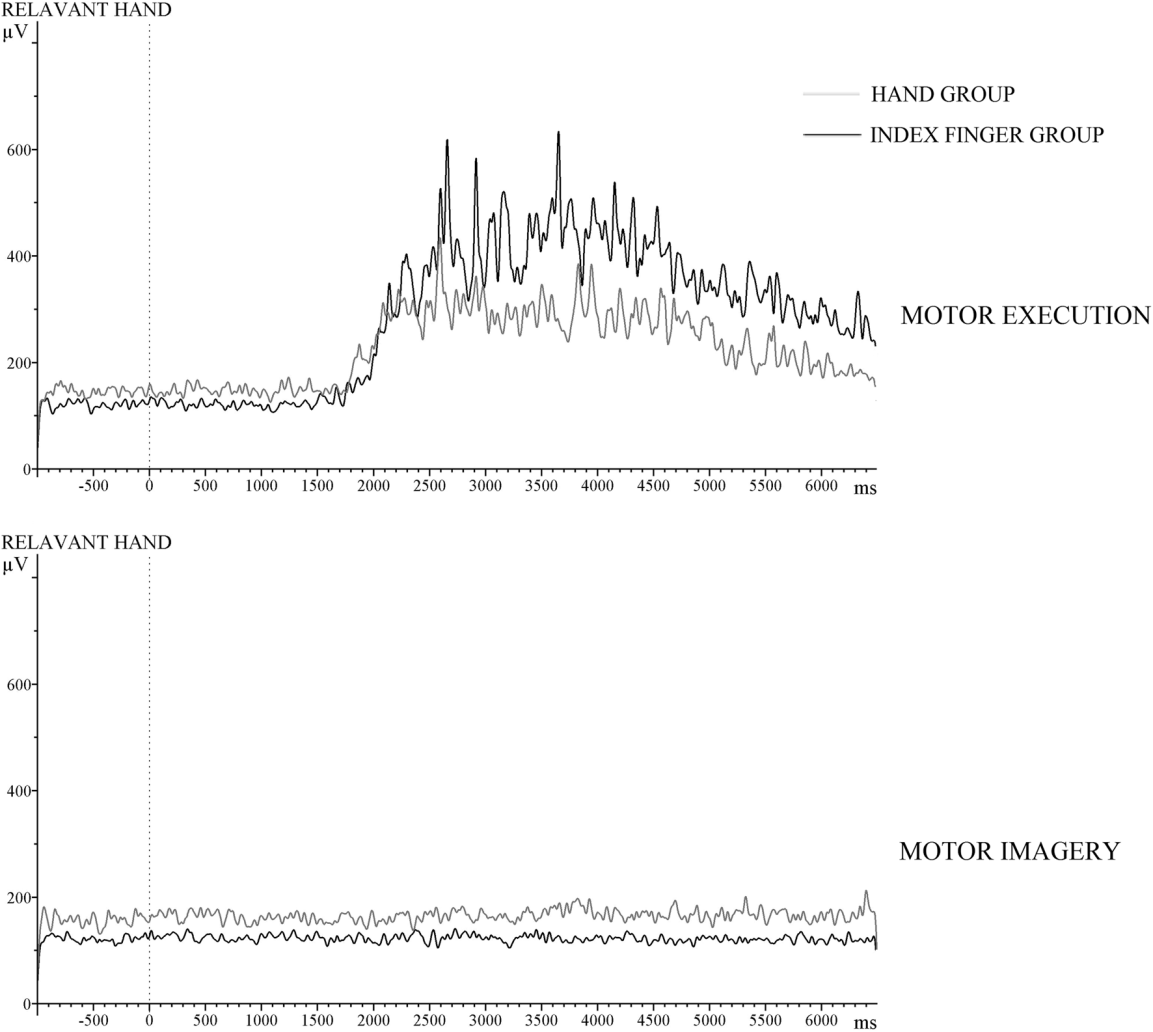
Outcome of the wavelet analysis performed on the raw EMG signal measured from the electrodes attached to the left and right forearms in the training phase. The grand averages are only presented for the relevant hands for the motor execution and motor imagery conditions from − 1000 ms before the Go/NoGo signal (0 ms) to 6000 ms

Muscular activity was examined in the training phase to check whether participants followed the instruction not to move their fingers in the case of motor imagery. In Fig. 8, muscular activity is presented for relevant hand, separately for the average of trials that required motor execution and motor imagery for each group. No main effect of Group was observed, *p* = 0.29. No significant effect of Block was observed, *F*(6,132) = 2.78, *ϵ* = 0.39, *p* = 0.06, *η*_*p*_^2^ = 0.11. A significant difference was observed as a function of Type of Sequence, *F*(1,22) = 117.12, *p* < 0.001, *η*_*p*_^2^ = 0.84. These results indicate that participants contracted their muscles according to the required motor task, i.e., during motor execution, and not during motor imagery. A significant interaction between Type of Sequence and Group was also observed, *F*(1,22) = 10.44, *p* < 0.004, *η*_*p*_^2^ = 0.32. Inspection of Fig. 8 shows larger muscular activity in the index finger than in the four fingers group. No significant difference was observed as a function of EMG channel, *F*(1,22) = 0.11, *p* = 0.74, *η*_*p*_^2^ = 0.005; and no significant interaction between EMG channel and Group was observed, *F*(1,22) = 0.94, *p* = 0.34, *η*_*p*_^2^ = 0.04.

## Discussion

In this study, we questioned how effector specific the effect of sequence learning by motor execution and motor imagery is by varying the execution mode during a training and a test phase. We wanted to establish whether during learning a sequential motor skill a representation at a motor level develops that is muscle specific and, therefore, effector dependent instead of a representation at a cognitive level that contains spatio-temporal characteristics and is effector independent (see Verwey 2016). Based on the study of Keele et al. (1995), it may be hypothesized that motor skill learning is rather effector independent. On the other hand, several studies showed that the execution of motor sequences becomes increasingly effector dependent with extensive practice (Hikosaka et al. 1999; Bapi 2000; Verwey 2001; Verwey and Wright 2004). First, we addressed the question how effector specific the effect of motor execution on sequence learning is. Second, we discussed whether sequence learning by motor imagery is effector dependent or not.

First, we were interested how effector specific the effect of sequence learning by motor execution is by varying the execution mode among participants. Behavioral results from the training phase showed a reduction in RT and an increase in PC. The reduction in RT mainly concerned the reduction of the initiation time (pressing the first key of a sequence). Learning effects obtained from the test phase revealed that only motor execution for keys from 2 to 5 in the first block of the test phase depended on the execution mode used in the training phase (i.e., participants who practiced with an index finger in the training phase, executed the sequences faster with an index finger in the test phase). These results indicate aspecific learning effects of execution mode used in the training phase by participants from the index finger group, but all other results (from the first, but also from the second block of the test phase) did not depend on group, which suggests that sequence-specific learning effects are effector independent. Our results from the test phase seem to reflect the learning of a spatio-temporal sequence rather than the learning of a specific motor pattern in both groups. Thus, at this level learning a sequential skill seems not muscle specific, which replicates several previous studies (Keele et al. 1995; Grafton 1998). Thus, the structure of a motor sequence has been reinforced more at a cognitive level (what is related with a motor program) than at a motor level (Frank et al. 2014; Verwey 2016). It has been proposed that with relatively limited practice spatial representations develop that are effector independent, while the development of motor representations (being effector dependent) is related with an extensive practice (Verwey 2016). For example, in the study of Verwey and Wright (2004), in which two components were developed (the effector-dependent and the effector-independent components), the number of sequences which were practiced was much larger than in our experiment (i.e., 1750 sequences in the first day of practice, whereas in our study the first day of practice consisted only of 400 sequences). The number of practiced sequences could play a relevant role in the development of the effector-dependent component.

Second, we examined how effector specific the effect of motor imagery on motor learning is. Behavioral results from the test phase revealed that participants responded faster and more accurately while executing familiar imagined sequences relative to unfamiliar sequences, showing that motor imagery is beneficial during the acquisition of motor skills. Interestingly, no significant difference between the index finger group and the hand group was observed. These results indicate that learning by motor imagery is also effector independent. Similar to the motor learning with motor execution, the fact that motor imagery is effector independent suggests the development of a spatio-temporal pattern rather than of the specific motor pattern. In other words, results from the test phase observed in the case of motor learning with motor imagery also revealed that the structure of motor sequence representation has been reinforced more at a cognitive level instead of motor level. The question remains open whether more practice with motor imagery could also result in the development of motor representation, which has been shown to be effector dependent (Hikosaka et al. 1999; Park and Shea 2003; Verwey and Wright 2004). Our results are in accordance with the study performed by Mizuguchi (2014), showing that activation of brain regions during motor imagery is effector independent. Similar brain activation has been found during motor imagery while imaging an extension and a flexion of right/left hand and right/left foot (Mizuguchi 2014), i.e., the left supplementary motor area and inferior frontal gyrus/ventral premotor cortex. Future research needs to clarify whether comparable results can be obtained when learning a motor skill with motor execution and motor imagery with other effectors, i.e., the arms or legs.

In this study, we demonstrated that motor skill learning with motor execution and with motor imagery is effector independent, indicating the development of spatial representation of a motor sequence. These results can be explained by several underlying neural mechanisms for motor skill learning (Hikosaka et al. 2002). Based on Hikosaka’s model (2002), two cortical systems are activated during learning a motor sequence (represented in two ways: spatial and motor), i.e., cortex–basal ganglia and cortex–cerebellum loop circuits. According to this model, spatial sequences are supported by parietal–prefrontal cortical loops (relying on attention and working memory), and these sequences are effector independent. On the other hand, motor sequences are supported by premotor–motor cortical loops, and these sequences are effector dependent. It has been postulated that processing of spatial sequence occurs earlier during acquisition of a motor skill than processing of motor sequence, which requires long-term practice, what is in line with previous findings (Hikosaka et al. 1999; Bapi 2000; Verwey 2001; Verwey and Wright 2004). Our results suggest that this model may explain both motor skill learning with motor execution, and motor skill learning with motor imagery as we observed the learning of a spatio-temporal sequence rather than the learning of a specific motor pattern in both groups. As we mentioned above, more practice might be needed to induce effector-dependent learning, indicating the activation of premotor–motor cortical loops which is related to the development of motor sequence.

In the current study, better motor performance was observed for previously executed and imagined sequences as compared with unfamiliar sequences, indicating sequence-specific learning effects. These results replicate the learning effects that we found in our previous studies (Sobierajewicz et al. 2016, 2017). In the case of learning a sequence of movements, it is important to differentiate sequence-specific learning which is based on sequence-specific representations at the central and motor processing level (Verwey 2015) from sequence-aspecific learning which is more related with an improved ability to decode stimuli or familiarization with the task (e.g., keeping the fingers in a suitable posture), etc. (Sobierajewicz et al. 2017). In the current study, behavioral results revealed significant differences only in the first block of the test phase while executing familiar imagined, familiar executed, and unfamiliar sequences. These results are in line with our previous findings showing that both motor execution and motor imagery induce sequence-specific learning (Sobierajewicz et al. 2016, 2017). The absence of significant differences in the second block of the test phase may be explained by the fact that unfamiliar sequences were learned by participants of both groups in the first block of the test phase.

In our analyses, we could also observe that using a different execution mode in the training phase has influence on particular processing phases of sequence skill. During the execution of a motor skill, three processing phases of sequence skill can be distinguished: an initiation phase, a concatenation phase, and an execution phase (Abrahamse et al. 2013). The first phase, an initiation, is related to the selection and preparation of the sequence. The key presses following sequence initiation are typically much faster involving only execution processes. Our results from the training phase revealed that the time needed to initiate a sequence was, indeed, longer for the index finger group than for the hand group. These results for the first key press are in line with the notion that the first key press is typically much slower than the subsequent key presses (Verwey 1999). The significant difference between groups in initiation might be explained by the fact that participant in the index group had to prepare moving their index finger to particular keys, which could slow down the first key press. Interestingly, our results from the training phase revealed that there was no significant difference between groups involving execution processes. Thus, our results show that the execution mode influenced the initiation phase, but not the execution phase during learning a required motor task.

Our findings showed that motor skill learning with motor execution and motor imagery is effector independent. However, one might argue that in the present study the statistical power was too low. Null results are not easy to interpret. A potential limitation of this study arises from the fact that the number of participants was not enough to demonstrate effector-dependent effects. On the other hand, no trend effects were present; therefore, we favor the interpretation that in our study, the sequential fine motor skill was acquired in an effector-independent manner.

In conclusion, our results demonstrate that learning a fine sequential motor skill by motor execution is effector independent, which extends previous findings to the Go/NoGo DSP task. Importantly, we were also able to demonstrate that learning a motor skill with motor imagery is also effector independent. In both cases of learning (i.e., either with motor execution or motor imagery), results suggest the development of spatio-temporal representations rather than muscle-specific representations.

## Electronic supplementary material

Below is the link to the electronic supplementary material.

Supplementary material 1 (DOCX 124 kb)
