# Supplementary material for: How effector-specific is the effect of sequence learning by motor execution and motor imagery?
Source: Exp Brain Res. 2017 Sep 30;235(12):3757–69. doi: 10.1007/s00221-017-5096-z (PMC5671521; doi:10.1007/s00221-017-5096-z)

Appendix

Sequences of five key presses used in the experiment:

6 structures of the sequence, 4 versions each

1-**a**, 2-**s**, 3-**d**, 4-**f**

1-**;**, 2-**l**, 3-**k**, 4-**j**


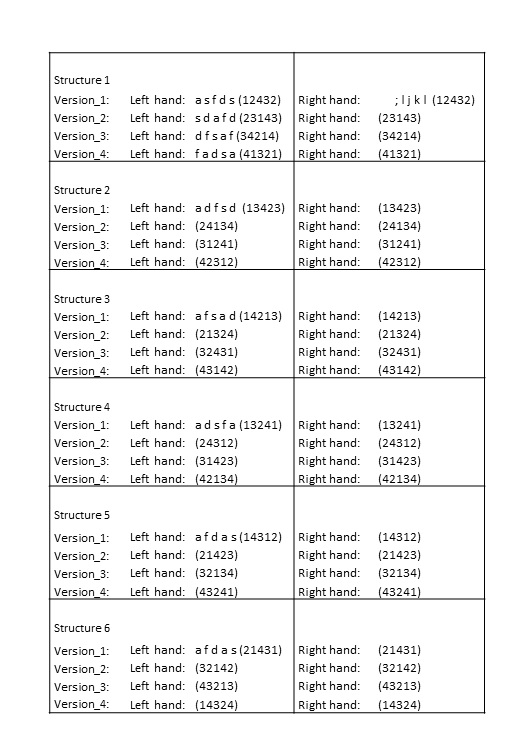

Supplement: Supplementary file 1 — Supplementary material 1 (DOCX 124 kb) [file 221_2017_5096_MOESM1_ESM.docx]
